# Does Psychological Quality Matter? A Moderated Model of Family Screen Exposure and Academic Burnout Among Early Adolescents

**DOI:** 10.3390/bs16101919

**Published:** 2026-10-16

**Authors:** Huiwen Yong, Xianghui Yan, Linlin Sun, Keyu Li, Qinglian Wu, Yichun Mai, Xin Wang

**Affiliations:** 1 School of Education and Psychology, Southwest Minzu University, Chengdu 610225, China; 2 Sichuan Minzu Education Development Research Center, Southwest Minzu University, Chengdu 610225, China

**Keywords:** academic burnout, problematic family screen use, psychological quality, adaptability, moderation, early adolescence

## Abstract

Drawing on Conservation of Resources (COR) theory, this study examined whether psychological quality and its three dimensions condition the association between problematic family screen use and academic burnout among early adolescents, and whether the wider sampling setting further conditions this association. A total of 1399 fifth- and sixth-grade students were recruited by cluster sampling from two provincial settings in south-west China. Validated scales measured problematic family screen use (9 items), academic burnout (16 items), and multidimensional psychological quality (26 items; cognitive characteristics 7, personal qualities 8, and adaptability 11). Hierarchical regression and conditional process analyses were used to examine the relationships of interest. Problematic family screen use was positively associated with academic burnout (r = 0.45, *p* < 0.001). The moderating role of psychological quality was specific to particular components rather than uniform across the construct. Adaptability buffered the association, and the conditional effect of screen use fell from 0.88 at low adaptability to 0.04 at high adaptability (interaction B = −0.044, *p* < 0.001, ΔR^2^ = 0.047). By contrast, the composite score and the cognitive and personal dimensions amplified the association (B = 0.007, 0.066, and 0.059, respectively), although the composite effect was small (ΔR^2^ = 0.004). Adaptability was also the only component for which the moderating effect differed between the two sampled settings (three-way B = −0.019, *p* = 0.020), whereas the corresponding three-way term for the composite score was not significant (*p* = 0.154). Academic burnout was higher in Yunnan than in Sichuan (*p* < 0.001) and did not differ between boys and girls (*p* = 0.872). Because the design is cross-sectional, these findings describe conditional associations rather than causal effects. The results caution against treating psychological quality as a single protective resource: adaptability was the component most likely to offset, and the cognitive and personal components the components least likely to offset, the association between problematic screen use and academic burnout.

## 1. Introduction

In an era characterized by rapid AI proliferation and deep educational digitalization, screen-based devices have transformed from mere entertainment tools into pervasive developmental niches that actively shape children’s learning, socialization, and neuro-cognitive development (Super & Harkness, 1986; T. Li, 2021). Unlike previous technological transitions, contemporary digital environments constitute “always-on” ecologies that blur boundaries between leisure, education, and social obligation, creating unprecedented demands for continuous psychological adaptation (Reinecke & Hofmann, 2016). Contrary to deterministic narratives positing linear relationships between screen time and developmental outcomes (Twenge & Farley, 2021), emerging evidence suggests that the psychological impact of digital exposure is contingent upon complex person-environment interactions (Lee et al., 2022). This complexity is particularly salient among upper elementary students (fifth and sixth graders), who face the dual challenges of pre-adolescent neuro-developmental transitions and intensifying academic pressures associated with the elementary-to-secondary school transition (Schuurmans et al., 2022).

Academic burnout, a psychological syndrome encompassing emotional exhaustion, academic disengagement, and reduced sense of accomplishment (Hwang & Kim, 2022), represents a critical yet understudied outcome in this developmental window. While public discourse frequently attributes adolescent burnout to quantitative screen exposure (X. Li et al., 2025) and lower well-being (Molina Moreno et al., 2025), such surface-level assessments overlook the qualitative nature of digital demands: the chronic activation of stress response systems without corresponding recovery periods (Edwards et al., 1998). We propose that problematic screen use functions not merely as a time-based risk factor, but as a demand–resource imbalance in which individual psychological resources play a decisive role in determining the severity of burnout.

### 1.1. Problematic Family Screen Use as Chronic Resource Erosion

Contemporary conceptualizations of screen exposure require evolution beyond quantitative metrics toward qualitative assessments of “problematic screen use”, a dependent, uncontrolled behavioral pattern characterized by functional interference in daily life (Domoff et al., 2019; H. Li et al., 2022). From the perspective of Conservation of Resources (COR) theory (Hobfoll, 1989), problematic family screen use represents a chronic resource erosion process. Excessive sensory stimulation induces cognitive overload, depleting attentional resources (Uncapher & Wagner, 2018); nocturnal blue light exposure disrupts sleep architecture, obstructing physiological recovery (Hale & Guan, 2015); and online interactions increasingly displace real-world social support systems, undermining critical psychological resources (Twenge & Campbell, 2018).

However, COR theory also posits that resource loss is not inevitable if individuals possess sufficient “resource caravans” (Hobfoll et al., 2018) to buffer against environmental stressors. In digital contexts, screen devices create a net drain on resources, but the extent to which this drain translates into academic burnout may depend on the individual’s capacity to regulate and recover. Unlike acute stressors that mobilize immediate coping, the insidious nature of problematic screen use creates low-grade chronic demands that gradually erode regulatory capacities. Consistent with this resource-depletion logic, a 7-day daily diary study by Goh and Hartanto (2026) provided longitudinal evidence showing that daily problematic smartphone use prospectively predicts reduced self-control capacity, supporting the argument that problematic digital engagement can deplete the cognitive resources underlying self-regulation, with no significant reverse cross-lagged effect observed. This study argues that the critical question is not merely whether screen use is associated with burnout, but for whom and under what conditions this association is stronger or weaker.

### 1.2. Psychological Quality as a Candidate Moderator

Chinese developmental researchers have proposed the construct of psychological quality, named psychological suzhi (Nie et al., 2020), a culture-specific conceptualization denoting protective psychological resources enabling adolescents to navigate stress, synthesizing elements from Western constructs of resilience and well-being (Zhang, 2003; Zhang et al., 2017). This multidimensional construct comprises cognitive characteristics, personal qualities, and adaptability. L. Wu et al. (2018) have already shown that psychological suzhi can operate as a moderator; so, the present study does not claim that moderating role as a novel contribution; what it adds is a simultaneous test of the total score and of each dimension, together with a regional context variable.

Two competing predictions can be derived about the direction of moderation. According to the Stress-Buffering Model (Hollister-Wagner et al., 2001), high levels of protective factors weaken the link between stressors and strain, which implies that the association between screen use and burnout should be weaker among students with higher psychological quality. The Adaptive Calibration Model (Del Giudice et al., 2011) offers a different prediction: organisms calibrate their stress-response systems based on environmental demands, and highly responsive individuals may sustain effort in the face of persistent demands for longer than others. On this reading, the association between demand and strain could be stronger rather than weaker at higher levels of psychological quality. Because the two frameworks make opposite predictions, the present study treats the direction of moderation as an open empirical question rather than a directional hypothesis. Crucially, because psychological quality is multidimensional, the two predictions need not hold for every component at once: some components may buffer while others amplify, and a test that reports only the composite score would conceal such a dissociation.

### 1.3. Ecological Context: The Two Sampled Settings

The interaction between screen use and psychological quality does not occur in a vacuum; it is embedded within broader ecological contexts. Extending these frameworks through Developmental Niche Theory (Super & Harkness, 1986), we treat the wider sampling setting as a macro-level contextual variable. The present study sampled students in two provincial settings in south-west China, and these settings differ in educational culture, academic competition intensity, and familial digital governance patterns. Because each setting is represented by a single province, the analysis contrasts these two settings rather than two classes of cities, and no conclusion is drawn about first-tier or non-first-tier cities in general.

In highly competitive educational ecologies, screen use becomes closely linked to academic goal attainment through cybernetic loops of expectation and compliance (Edwards, 2008). Conversely, in settings where offline alternatives are less available, screen devices may serve as primary developmental stimuli with less parental mediation. Resource Caravans and Passageways theory (Hobfoll et al., 2018) suggests that the availability of ecological resources conditions the effectiveness of individual resources. Therefore, the moderating effect of psychological quality on the screen use-burnout association may vary across the two sampled settings.

### 1.4. Aims of the Present Study

**Figure 1:**
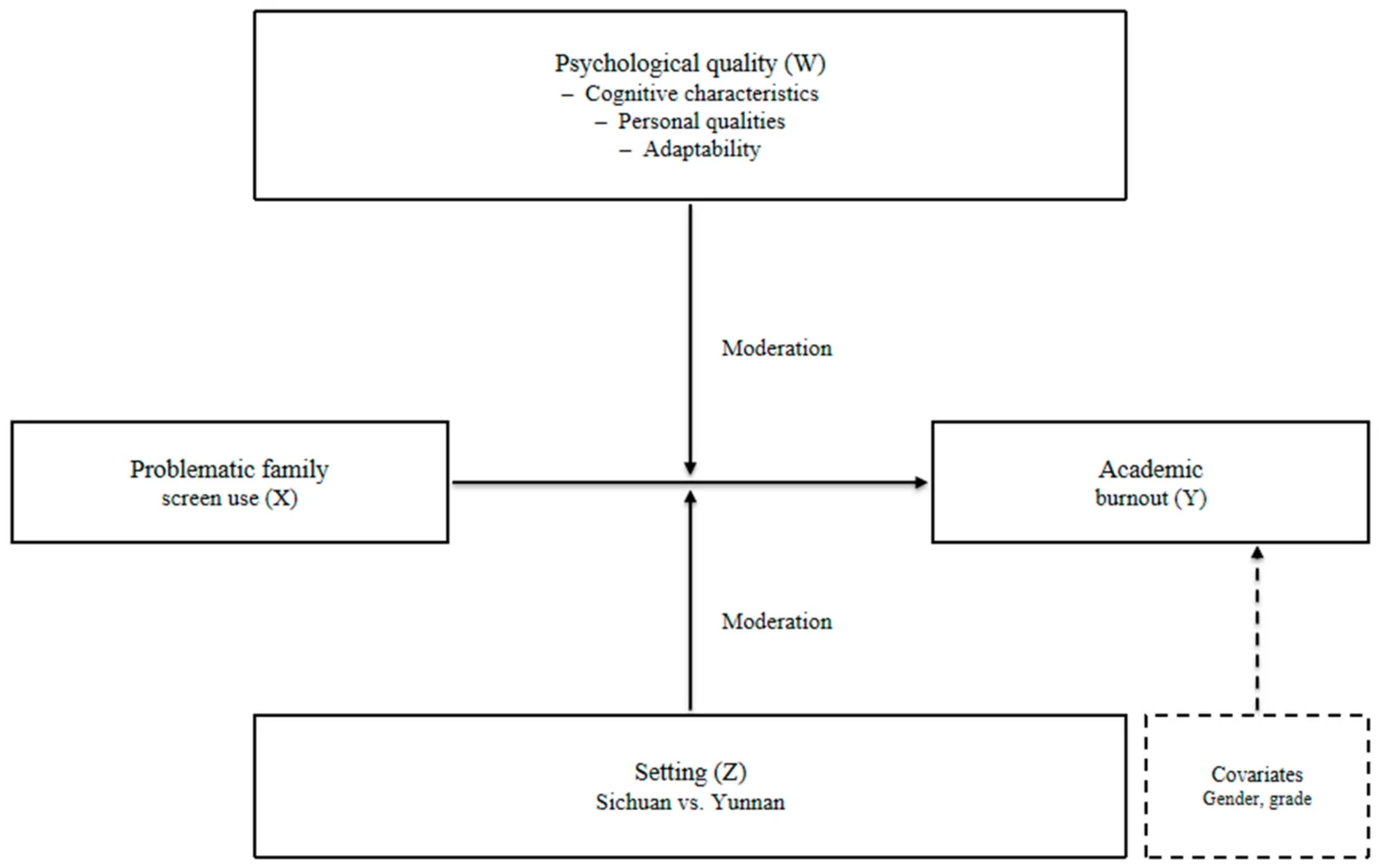
Conceptual model of the moderation of the association between problematic family screen use and academic burnout. The solid horizontal arrow is the main-effect path from problematic family screen use (X) to academic burnout (Y). Psychological quality (W) and its three dimensions (cognitive characteristics, personal qualities, and adaptability) moderate this path. The sampling setting (Z; Sichuan vs. Yunnan) further moderates the moderating effect of adaptability. Gender and grade are entered as covariates.

Despite these theoretical advancements, critical gaps persist. First, existing research has often treated psychological quality as an outcome or a mediator, and its moderating role has been examined in only a limited number of studies. Second, the direction of its moderating role is theoretically contested, and competing predictions have rarely been tested against each other. Third, few studies have examined how macro-level contextual variables interact with individual psychological characteristics to shape digital developmental outcomes, and fewer still have asked whether the components of a composite protective resource behave in the same way as the composite itself. Each of these gaps is addressed directly by the design described below: the first by treating psychological quality as a moderator, the second by specifying buffering and amplification as competing hypotheses, and the third by including the sampling setting as a contextual moderator and by testing the total score and each dimension separately. The hypothesized model is summarized in Figure 1.

To address these gaps, the present study investigates a moderated model wherein problematic family screen use is associated with academic burnout, and this relationship is moderated by psychological quality (including its dimensions) and by the wider sampling setting. Specifically, we hypothesize that:

**H1.**  *Problematic family screen use is positively associated with academic burnout.*

**H2.**  *Psychological quality moderates this association. Two competing predictions are tested: H2a, that the association is weaker at higher psychological quality (buffering), and H2b, that it is stronger (amplification).*

**H3.**  *The direction of moderation is examined separately for the three dimensions of psychological quality, without predicting a priori whether any one dimension will behave differently from the others.*

**H4.**  *The wider sampling setting further moderates these associations, and the analysis is presented as a contrast between the two sampled settings.*

## 2. Materials and Methods

### 2.1. Design and Participants

The study used a cross-sectional survey design. Because all variables were measured at a single time point, no causal or temporal claim is made anywhere in this paper; the analyses describe associations and the conditions under which they are stronger or weaker. This constraint applies to every result reported below.

A cluster sampling procedure was employed to recruit fifth- and sixth-grade students from ordinary primary schools in two provincial settings in south-west China. Written informed consent was obtained from both participants and their parents or guardians prior to data collection. A total of 1500 questionnaires were distributed, and 1399 complete and valid responses were retained (an effective response rate of 93.3%). Independent-samples t-tests and chi-square tests were conducted to examine potential systematic attrition by comparing the retained and the excluded questionnaires on the demographic variables; no significant differences were found (all *p*s > 0.05), which does not by itself establish that the data were missing completely at random.

The analyzed sample comprised 650 boys and 749 girls; 642 fifth-graders and 757 sixth-graders; and 698 students from the first sampled setting (Sichuan) and 701 from the second (Yunnan). In subsequent analyses, gender (0 = boys, 1 = girls), grade (1 = fifth grade, 2 = sixth grade), and sampling setting (1 = Sichuan, 2 = Yunnan) were included as control variables. The analysis file carries no school or class identifier; so, the models do not adjust for the clustering of students within classes and this is reported as a limitation in Section 4.6.

### 2.2. Measures

#### 2.2.1. Problematic Family Screen Use

Problematic family screen use was assessed using the Chinese version of the Problematic Media Use Measure-Short Form (PMUM-SF; Domoff et al., 2019) together with family-context items on conflict about device use. The scale contains 9 items evaluating problematic screen use within family contexts. Items were rated on a 5-point Likert scale ranging from 1 (strongly disagree) to 5 (strongly agree) and were scored in the direction of greater problematic use, so that higher scores indicate more severe problematic family screen use. In the present study, Cronbach’s alpha was 0.83.

#### 2.2.2. Psychological Quality

Psychological quality was measured using the Brief Psychological suzhi Scale for Primary School Students (L. Wu et al., 2018; Zhang et al., 2017). The scale includes 26 items covering three dimensions: cognitive characteristics (7 items), personal qualities (8 items), and adaptability (11 items). All items were scored on a 5-point Likert scale, with higher scores representing higher levels of psychological quality. In this study, Cronbach’s alpha was 0.88 for the total scale, and 0.76, 0.79, and 0.81 for the cognitive, personal, and adaptability subscales, respectively.

#### 2.2.3. Academic Burnout

Academic burnout was assessed using an adapted version of the Adolescent Student Burnout Inventory (Y. Wu et al., 2010), revised to be suitable for upper elementary students. The scale contains 16 items covering three dimensions: emotional exhaustion, academic alienation, and reduced sense of accomplishment. Seven positively worded items were reverse-scored so that higher total scores indicate more severe academic burnout. Cronbach’s alpha in the current sample was 0.81.

### 2.3. Data Screening

Responses were screened against the 1–5 response format. The analyzed file contained 1399 complete cases, each with an in-range response on all 51 items; no value fell outside the 1–5 response format and no item-level value was missing in the analysis file. Item-level missing values in the returned questionnaires had been imputed during data cleaning, and out-of-range responses had been replaced by the item mean (Table S1).

Common method bias (CMB) was examined using Harman’s single-factor test (Podsakoff et al., 2003) in SPSS 27.0. Unrotated principal component analysis of all 51 items yielded eight factors with eigenvalues greater than 1, and the first factor accounted for 22.35% of the total variance, below the conventional 40% threshold. This test is a weak diagnostic, and because the scales were strongly intercorrelated the likely presence of a general response-style component is addressed in the limitations.

### 2.4. Procedure and Analysis

Prior to formal survey administration, item order was randomized across scales to reduce response bias. Trained research assistants collected data in classrooms under standardized instructions. Participants were informed of the study’s purpose, anonymity, and confidentiality, and completed questionnaires independently.

Descriptive statistics, Pearson correlations, group comparisons, and reliability analyses were conducted in SPSS 27.0. Moderation was tested with the PROCESS macro (version 4.1; Model 1; Hayes, 2022) in which the predictor was problematic family screen use and the moderator was either the total psychological quality score or one of its three dimensions. Sampling setting, grade, and gender were entered as covariates. The moderator was treated as a continuous variable throughout; group-based values were used only to display conditional effects, which are reported at the 16th, 50th, and 84th percentiles of the moderator and, for ease of interpretation, at plus or minus one standard deviation. The three dimensions were tested in separate models rather than entered simultaneously, because they correlate 0.41 to 0.76 with one another and 0.80 to 0.85 with the total score, which would make a joint model unstable. A three-way interaction model (PROCESS Model 3) added the sampling setting as a second moderator. Conditional effects are reported with 95% confidence intervals. Unless otherwise stated, coefficients are unstandardized and all statistical tests are two-tailed.

## 3. Results

### 3.1. Descriptive Statistics, Reliability, and Correlations

Means, standard deviations, and observed ranges are reported in Table 1, the correlation matrix in Table 2, and reliability coefficients in Table 3. Problematic family screen use correlated positively with academic burnout (r = 0.45, *p* < 0.001), and the total psychological quality score correlated negatively with academic burnout (r = −0.24, *p* < 0.001) and with screen use (r = −0.51, *p* < 0.001). The three dimensions, however, did not behave alike. Cognitive characteristics were essentially unrelated to burnout (r = −0.005, *p* = 0.841); personal qualities were positively related to burnout (r = 0.13, *p* < 0.001); and adaptability was negatively related to burnout (r = −0.51, *p* < 0.001).

### 3.2. Group Differences in Academic Burnout

As shown in Table 4, students in Yunnan scored significantly higher on academic burnout than students in Sichuan (*p* < 0.001), and the difference was moderate in size. Boys and girls did not differ (*p* = 0.872), and the associated effect size was negligible.

### 3.3. Moderation by Overall Psychological Quality

Table 5 reports the moderation models. For the total psychological quality score, the interaction with problematic family screen use was positive and statistically significant, B = 0.007, SE = 0.002, t = 3.08, *p* = 0.002, but the increment it produced was very small, ΔR^2^ = 0.004, F(1, 1392) = 9.47. The conditional effect of screen use on burnout increased slightly across the range of the total score, from 0.55 at the 16th percentile (a score of 80) to 0.79 at the 84th percentile (a score of 114; Table 6). The direction is therefore one of amplification, consistent with H2b and inconsistent with H2a, but the effect is weak and should not be overstated.

**Figure 2:**
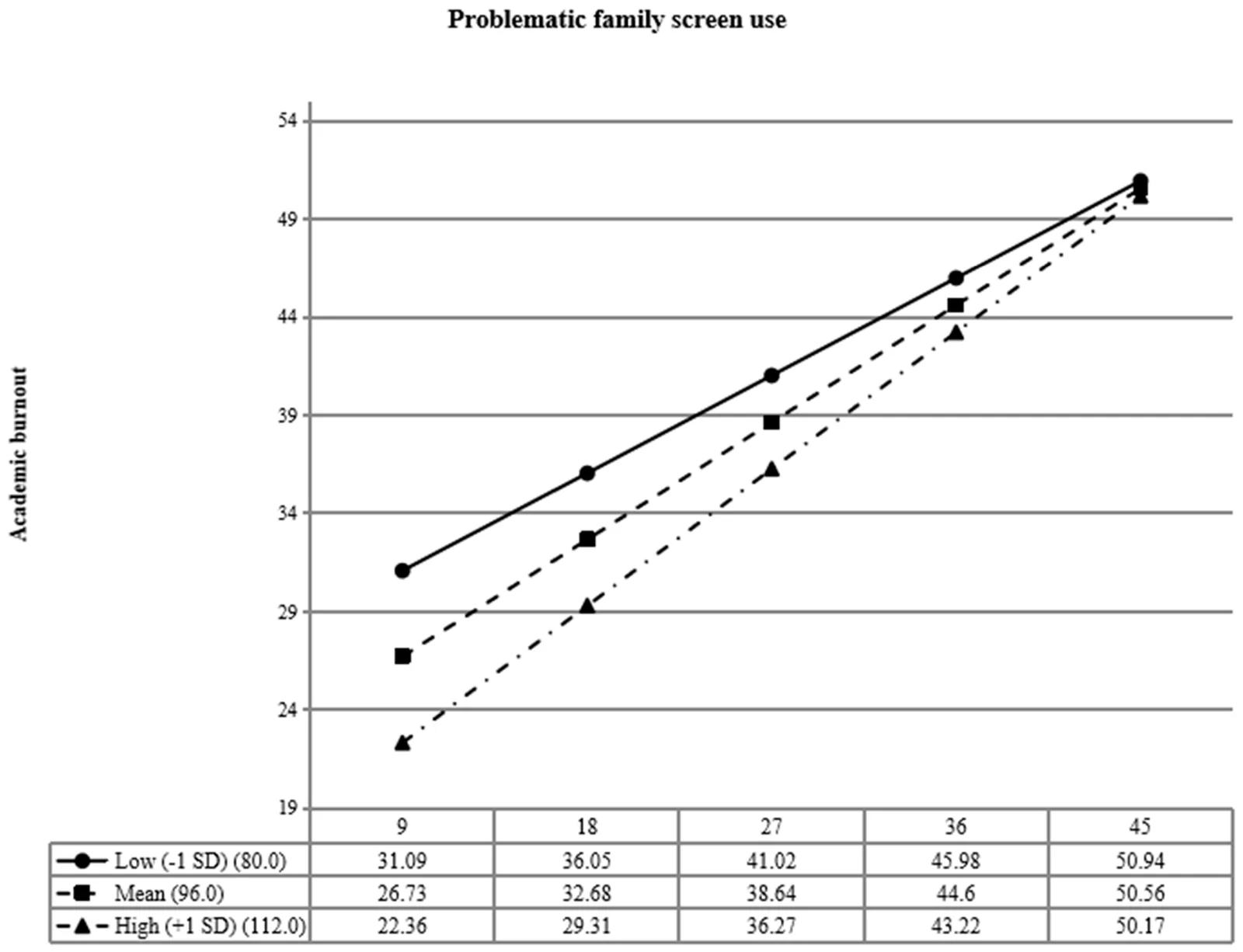
Simple slopes of problematic family screen use predicting academic burnout at low, mean, and high levels of total psychological quality.

Figure 2 displays the corresponding simple slopes. The gradient steepens modestly as the total score rises, which reproduces the pattern just described, and the three lines remain clearly separated, indicating that the association is positive at every level of the composite.

Figure 2 presents the conditional effect of problematic family screen use on academic burnout across three levels of the total psychological quality score. The slopes were 0.55, 0.65, and 0.79 at the low, median, and high levels, respectively. The gradient steepens only slightly, consistent with the small ΔR^2^ reported above; the horizontal axis plots raw scores of problematic family screen use and the vertical axis shows raw academic burnout scores.

### 3.4. Moderation by the Three Dimensions

**Figure 3:**
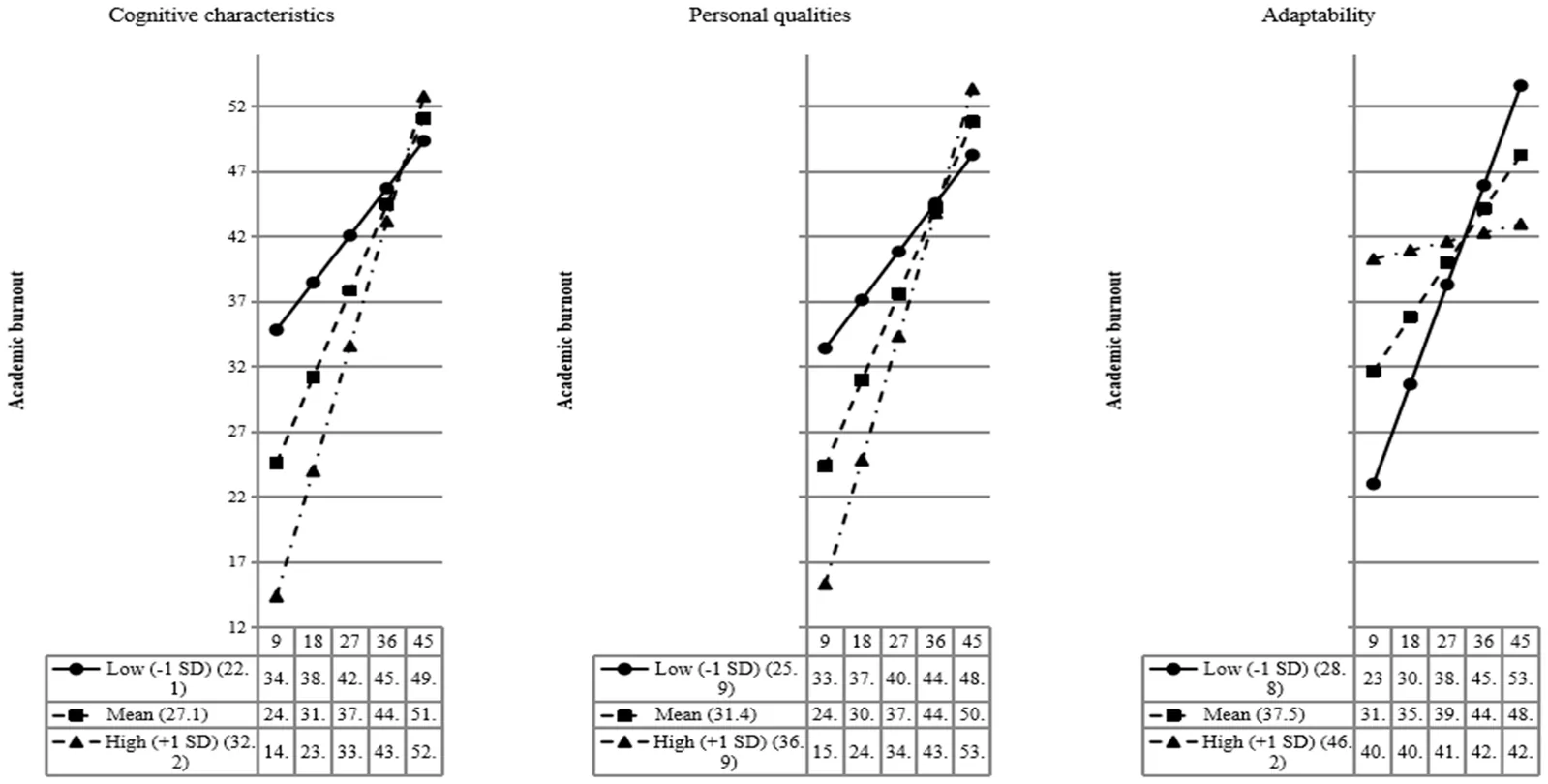
Simple slopes of problematic family screen use predicting academic burnout at low, mean, and high levels of each dimension of psychological quality (cognitive characteristics, personal qualities, and adaptability).

When the three dimensions were examined separately, a clear dissociation emerged (Table 5 and Figure 3). Cognitive characteristics (B = 0.066, *p* < 0.001) and personal qualities (B = 0.059, *p* < 0.001) amplified the association between screen use and burnout, and the increments they produced were substantial, ΔR^2^ = 0.047 and 0.049, respectively. Adaptability behaved in the opposite way: its interaction was negative (B = −0.044, *p* < 0.001, ΔR^2^ = 0.047), and the conditional effect of screen use fell from 0.88 at the 16th percentile to 0.04 at the 84th percentile, where the slope was no longer distinguishable from zero (Table 6). Adaptability therefore buffered the association in the sense of the Stress-Buffering Model, whereas the cognitive and personal dimensions amplified it in the sense of the Adaptive Calibration account. Because no directional prediction had been made for individual dimensions, this dissociation is an empirical outcome that qualifies H3.

Inspection of the three panels of Figure 3 shows that the buffering pattern is specific to adaptability: the lines converge and the slope flattens at high adaptability, whereas for cognitive characteristics and personal qualities, the lines diverge and the slope steepens as the dimension increases.

### 3.5. Moderation by Sampling Setting

We tested the three-way interaction of problematic family screen use, psychological quality, and sampling setting. For the total psychological quality score, the three-way term was not significant, B = −0.007, SE = 0.005, t = −1.43, *p* = 0.154, ΔR^2^ = 0.001. The same was true for cognitive characteristics (B = −0.015, *p* = 0.221) and for personal qualities (B = 0.000, *p* = 0.981). Only adaptability showed a significant three-way interaction, B = −0.019, SE = 0.008, t = −2.33, *p* = 0.020, ΔR^2^ = 0.002, F(1, 1389) = 5.42. H4 is therefore supported only for adaptability, not for the composite score.

**Figure 4:**
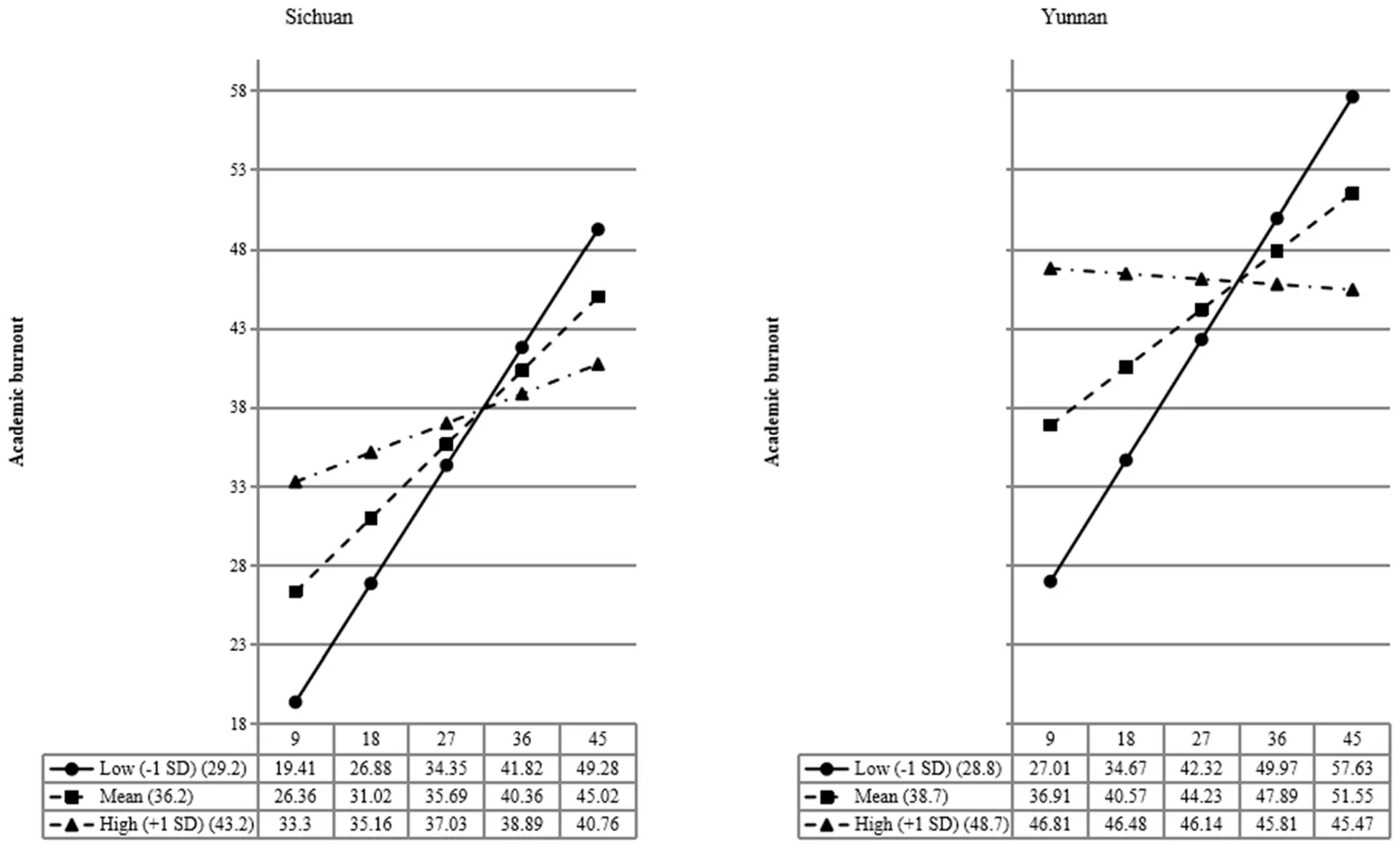
Simple slopes of problematic family screen use predicting academic burnout at low, mean, and high levels of adaptability, separately for the two sampled settings.

Table 7 reports the three-way model for adaptability, and Table 8 the conditional effects of screen use within each setting; Figure 4 displays the corresponding simple slopes. In both settings, the buffering pattern was present, and it was stronger in Yunnan: the conditional interaction between screen use and adaptability was −0.045 in Yunnan and −0.026 in Sichuan. Expressed as conditional slopes, screen use was unrelated to burnout at high adaptability in both settings (0.34 in Sichuan and −0.11 in Yunnan), and was most strongly related to burnout at low adaptability (0.84 in Sichuan and 0.75 in Yunnan).

### 3.6. Exploratory Analysis: Gender

Gender was tested as an additional moderator in an exploratory analysis. The interaction was not significant, B = 0.070, SE = 0.070, t = 0.99, *p* = 0.322, ΔR^2^ = 0.001. Boys and girls did not differ in the strength of the screen-use-burnout association, nor did they differ in mean burnout (Section 3.2). This result is reported for completeness, and no directional hypothesis was advanced for it.

## 4. Discussion

Building on Conservation of Resources (COR) theory and developmental contextual perspectives, the present study investigated a moderated model linking problematic family screen use to academic burnout among early adolescents, with a focus on the buffering versus amplifying role of psychological quality and its dimensions and on the contextual role of the wider sampling setting. Overall, H1 was supported; H2a was supported for adaptability and H2b for the composite score and for the cognitive and personal dimensions; H3 was qualified by a dimension-level dissociation; and H4 was supported only for adaptability.

First, problematic family screen use was positively associated with academic burnout, confirming H1. Second, the direction of moderation depended on which component of psychological quality was examined: adaptability buffered the association, whereas the composite score and the cognitive and personal dimensions amplified it, although the composite effect was small. Third, the buffering effect of adaptability, and only that effect, differed between the two sampled settings. Fourth, boys and girls did not differ, either in mean burnout or in the strength of the association. These findings qualify the common assumption that psychological resources necessarily protect against digital risk. Psychological quality does matter, but its components do not all matter in the same direction.

### 4.1. Problematic Family Screen Use and Academic Burnout

Consistent with COR theory (Hobfoll, 1989) and prior digital developmental research (Domoff et al., 2019; X. Li et al., 2025; Twenge & Campbell, 2018), problematic family screen use was associated with academic burnout. From a resource-erosion perspective, poorly regulated screen engagement depletes attentional resources, disrupts sleep recovery, and displaces supportive interpersonal interactions, creating a chronic state of resource drain (Uncapher & Wagner, 2018). Recent longitudinal daily-diary evidence further supports the resource-erosion perspective: Goh and Hartanto (2026) demonstrated that elevated problematic smartphone use predicted subsequent reductions in self-control capacity, with no meaningful reverse effect. The correlation observed here (r = 0.45) is moderate in size and is consistent with these accounts while remaining a cross-sectional association that cannot establish direction.

### 4.2. A Composite That Amplifies and a Dimension That Buffers

A stress-buffering interpretation would predict a weaker slope at higher psychological quality. For the composite score, the data show the opposite, whereas for adaptability, they show exactly the expected buffering pattern. Two readings are consistent with the amplification observed for the composite and for the cognitive and personal dimensions. The first is the over-accommodation account: students who are more capable of sustaining effort may continue to meet digital and academic demands for longer before disengaging, so that among them, the accumulated demand is more closely tied to reported burnout, whereas students lower in these characteristics may report elevated burnout largely independently of screen use. Work on perceived expectations to remain constantly available and productive is relevant here, as it documents that such expectations predict emotional exhaustion and feelings of inadequacy over time, and that burnout symptoms in turn predict increases in perceived expectations (Hartanto et al., 2025). That study used a longitudinal bidirectional design, whereas the present data are cross-sectional; so, the direction of the gradient cannot be resolved here. The second reading is statistical rather than substantive: when a moderator is strongly related to the outcome, the fitted interaction can reflect a floor effect at low moderator values rather than a genuine reinforcement process, and this possibility is particularly relevant for the composite score, whose interaction increment was very small. The present cross-sectional data cannot separate these accounts, and the amplifying gradient should be treated as a hypothesis for longitudinal work rather than as an established mechanism.

The buffering effect of adaptability admits a more straightforward interpretation. Adaptability items describe the capacity to adjust to changing circumstances, to tolerate frustration, and to regulate responses to difficulty, and the dimension was itself strongly and negatively related to burnout (r = −0.51). Students high in adaptability appear to convert the demands of problematic screen use into less burnout, exactly as the Stress-Buffering Model predicts for a functional protective factor. That the composite score did not inherit this pattern reflects the fact that its other two components worked in the opposite direction, and it illustrates why a total score can conceal component-specific processes.

### 4.3. Why the Dimension-Level Dissociation Matters

Because the dimensions behaved differently, the results argue against treating psychological quality as a single undifferentiated protective resource and against interpreting the composite score as if it summarized its components. The observed heterogeneity is accompanied, however, by a measurement caution. In exploratory factor analysis of the present data, cognitive characteristics and personal qualities did not separate into distinct factors; all seven cognitive and all eight personal items loaded on the same factor, and only the adaptability items formed a distinguishable group (Supplementary Table S19). Dimension-level conclusions from this instrument are therefore provisional, and the dissociation reported here should be replicated with measures that demonstrate discriminant validity among the dimensions. Researchers should be cautious about attaching distinct functional roles to the dimensions of this instrument without first establishing that the dimensions are empirically separable.

### 4.4. Sampling Setting and Gender

Students in Yunnan reported higher burnout than students in Sichuan, and the buffering effect of adaptability was stronger in Yunnan. Both findings are reported as differences between two sampled settings. No claim is made that the results generalize to provinces or cities that were not sampled, and no inference is drawn about first-tier or non-first-tier cities as such. The absence of a gender difference, both in mean level and in moderation, is consistent with the view that the screen-use-burnout association in this age group is not strongly gendered, although a null result of this kind also reflects the limited power of an interaction test.

### 4.5. Theoretical and Practical Implications

The results extend COR theory in a specific way: an individual resource that is negatively related to strain overall does not necessarily weaken the link between a chronic demand and strain, and a composite resource can contain components that operate in opposite directions. Resource-rich individuals may differ from resource-poor individuals in how tightly their strain tracks that demand, and which resource is examined changes the answer. Practically, the findings argue against assuming that raising psychological quality in general will neutralize the association between problematic screen use and burnout. Interventions that specifically strengthen adaptability, such as training in flexible coping and frustration tolerance, are better supported by these data than undifferentiated efforts to raise the composite score; and interventions that target the regulation of screen use itself remain necessary. Parental mediation and family digital rules appear to be more promising targets than the cultivation of individual strengths alone. These are hypotheses for intervention research, because the present study did not measure parental mediation or family digital rules.

### 4.6. Limitations and Future Directions

Several limitations should be acknowledged. First, the cross-sectional design precludes causal inferences; longitudinal designs are needed to clarify temporal dynamics and, in particular, to determine whether the amplifying gradient observed for the composite and for the cognitive and personal dimensions reflects over-accommodation or a floor effect at low moderator values. Second, all measures were self-reported, which may introduce a general response-style component; the strong correlations among conceptually distinct scales are consistent with such a component, and multi-informant and behavioral measures are needed. Third, the psychological quality instrument comprises 26 items whose cognitive and personal dimensions did not separate in factor analysis; so, dimension-level conclusions are provisional. Fourth, several items of that instrument concern the regulation of screen use, which means the predictor and the moderator are not fully independent in content; this overlap should be eliminated in future measurement work. Fifth, each setting was represented by a single province, and students were sampled in classes within schools; because the analysis file carries no school or class identifier, the standard errors are not adjusted for clustering and this could not be examined further. Sixth, the interaction increments for the composite score and for the three-way models were small; so, those particular effects should be interpreted with caution. Notably, Goh and Hartanto (2026) provided daily longitudinal evidence showing that problematic digital use may prospectively erode psychological resources such as self-control. However, our cross-sectional data cannot verify such temporal ordering; future longitudinal or daily diary designs are required to disentangle reciprocal relations between problematic family screen use and psychological resources.

Future research may investigate: (1) how specific types of screen use (educational vs. recreational) moderate these pathways; (2) neurocognitive correlates of over-accommodation and adaptability-based resource depletion; (3) the cross-cultural generalizability of the buffering-by-adaptability pattern; and (4) intervention studies targeting adaptive flexibility and mindful digital engagement.

## 5. Conclusions

Among the early adolescents surveyed, problematic family screen use was positively associated with academic burnout. The moderating role of psychological quality was not uniform. Adaptability buffered the association, so that screen use was unrelated to burnout among students high in adaptability, whereas the composite score and the cognitive and personal dimensions amplified it, although the composite effect was small. Adaptability was also the only dimension whose moderating effect differed between the two sampled settings. These conditional associations are cross-sectional and require longitudinal confirmation, but they caution against the assumption that strengthening psychological quality in general will by itself offset the association between problematic screen use and academic burnout; the component that appears to do the protective work is adaptability.

## Figures and Tables

**Table 1:** Descriptive statistics (N = 1399).

Variable	N	M	SD	Min	Max
Problematic family screen use	1399	22.31	7.95	9.00	45.00
Academic burnout	1399	49.86	11.63	23.00	80.00
Psychological quality (total)	1399	96.01	16.03	47.00	130.00
Cognitive characteristics	1399	27.15	5.06	11.00	35.00
Personal qualities	1399	31.38	5.48	13.00	40.00
Adaptability	1399	37.48	8.71	16.00	55.00

Note: N = number of valid cases; M = mean; SD = standard deviation; Min and Max are the observed extremes of the analyzed sample.

**Table 2:** Pearson correlations (N = 1399).

Variable	1	2	3	4	5	6
1. Problematic family screen use	1					
2. Academic burnout	0.453 ***	1				
3. Psychological quality (total)	−0.513 ***	−0.236 ***	1			
4. Cognitive characteristics	−0.358 ***	−0.005	0.849 ***	1		
5. Personal qualities	−0.271 ***	0.129 ***	0.803 ***	0.758 ***	1	
6. Adaptability	−0.565 ***	−0.513 ***	0.842 ***	0.504 ***	0.409 ***	1

Note: *** *p* < 0.001 (two-tailed). The correlation between personal qualities and academic burnout (0.129) and the near-zero correlation between cognitive characteristics and academic burnout (−0.005) are emphasized in the text.

**Table 3:** Internal consistency of the scales (N = 1399).

Scale	Items	Cronbach’s Alpha	Corrected Item-Total Range
Problematic family screen use	9	0.830	0.27–0.68
Academic burnout	16	0.806	0.13–0.66
Cognitive characteristics	7	0.760	0.38–0.52
Personal qualities	8	0.788	0.44–0.56
Adaptability	11	0.808	0.23–0.65
Psychological quality (total)	26	0.885	0.15–0.57

Note: alpha = Cronbach’s alpha; the final column shows the range of corrected item-total correlations.

**Table 4:** Academic burnout by sampled setting and gender (N = 1399).

Grouping	Level	*n*	M	SD	Test Statistic
Setting	Sichuan	698	46.56	12.18	t(1397) = −11.036, *p* < 0.001, d = −0.590
	Yunnan	701	53.14	10.04	
Gender	Boys	650	49.91	11.31	t(1397) = 0.161, *p* = 0.872, d = 0.009
	Girls	749	49.81	11.91	

Note: d = Cohen’s d. Setting 1 = Sichuan, Setting 2 = Yunnan; 0 = boys, 1 = girls. Levene’s test indicated unequal variances for the setting contrast (F = 17.89, *p* < 0.001) and equal variances for the gender contrast (F = 2.47, *p* = 0.116); the unequal-variance solution for the setting contrast was t(1345.75) = −11.032, *p* < 0.001.

**Table 5:** Moderation of the screen-use-burnout association by psychological quality and its three dimensions (N = 1399).

Model/Predictor	B	SE	t	*p*	ΔR^2^
(a) Total psychological quality					0.0044
Screen use (centered)	0.6619	0.0371	17.84	<0.001	
Psychological quality (centered)	−0.0267	0.0190	−1.40	0.161	
Screen use × Psychological quality	0.0069	0.0022	3.08	0.002	
(b) Cognitive characteristics					0.0472
Screen use (centered)	0.7352	0.0328	22.41	<0.001	
Cognitive characteristics (centered)	0.3145	0.0509	6.18	<0.001	
Screen use × Cognitive characteristics	0.0655	0.0062	10.56	<0.001	
(c) Personal qualities					0.0486
Screen use (centered)	0.7351	0.0312	23.57	<0.001	
Personal qualities (centered)	0.4403	0.0449	9.80	<0.001	
Screen use × Personal qualities	0.0587	0.0053	10.99	<0.001	
(d) Adaptability					0.0467
Screen use (centered)	0.4625	0.0342	13.54	<0.001	
Adaptability (centered)	−0.5923	0.0308	−19.23	<0.001	
Screen use × Adaptability	−0.0445	0.0039	−11.50	<0.001	

Note: Each block (a)–(d) is a separate model. Screen use and the moderator were mean-centered in every model; in addition to the terms shown, each model controlled for sampling setting, grade, and gender, and all coefficients are unstandardized. ΔR^2^ is the increment attributable to the interaction term after the covariates and both main effects had been entered; the corresponding F values are F(1, 1392) = 9.47, 111.58, 120.87, and 132.21 for models (a)–(d), respectively.

**Table 6:** Conditional effect of screen use on academic burnout at values of each moderator (N = 1399).

Moderator	Value	Conditional Effect of Screen Use	SE	t	*p*	95% CI
Total psychological quality	80 (16th pct.)	0.552	0.055	10.07	<0.001	[0.444, 0.659]
	95 (50th pct.)	0.655	0.038	17.48	<0.001	[0.582, 0.728]
	114 (84th pct.)	0.786	0.051	15.35	<0.001	[0.686, 0.886]
Cognitive characteristics	22 (16th pct.)	0.398	0.050	8.03	<0.001	[0.301, 0.495]
	28 (50th pct.)	0.791	0.032	24.47	<0.001	[0.728, 0.854]
	33 (84th pct.)	1.119	0.045	25.10	<0.001	[1.031, 1.206]
Personal qualities	26 (16th pct.)	0.419	0.046	9.02	<0.001	[0.328, 0.510]
	32 (50th pct.)	0.771	0.031	25.12	<0.001	[0.711, 0.831]
	37 (84th pct.)	1.065	0.039	27.49	<0.001	[0.989, 1.141]
Adaptability	28 (16th pct.)	0.884	0.055	16.02	<0.001	[0.776, 0.992]
	37 (50th pct.)	0.484	0.035	13.98	<0.001	[0.416, 0.552]
	47 (84th pct.)	0.039	0.045	0.88	0.382	[−0.048, 0.126]

Note: Values are the 16th, 50th, and 84th percentiles of the moderator. The conditional effect is the slope of screen use at that value. For adaptability, the slope at the 84th percentile is not significantly different from zero.

**Table 7:** Three-way interaction between screen use, adaptability and sampling setting (N = 1399).

Predictor	B	SE	t	*p*	ΔR^2^
Screen use (centered)	0.8554	0.1037	8.25	<0.001	0.0019
Adaptability (centered)	−0.7832	0.1046	−7.49	<0.001	
Screen use × Adaptability	−0.0070	0.0136	−0.51	0.610	
Setting (1 = Sichuan, 2 = Yunnan)	13.6147	1.7378	7.83	<0.001	
Screen use × Setting	−0.2663	0.0707	−3.77	<0.001	
Adaptability × Setting	0.0849	0.0645	1.32	0.188	
Screen use × Adaptability × Setting	−0.0191	0.0082	−2.33	0.020	
Grade	−2.7351	0.4749	−5.76	<0.001	
Gender (0 = boys, 1 = girls)	−0.7573	0.4351	−1.74	0.082	

Note: Setting was coded 1 = Sichuan, 2 = Yunnan. Screen use and adaptability were mean-centered. ΔR^2^ in the first row is the increment attributable to the three-way term, F(1, 1389) = 5.42, *p* = 0.020; the three-way terms for the total score, cognitive characteristics, and personal qualities were not significant (*p* = 0.154, 0.221, and 0.981, respectively). Because the setting is binary, the lower-order interaction terms that involve it refer to a setting value of zero, which lies outside the observed range, and are used only to construct the conditional effects in Table 8.

**Table 8:** Conditional effect of screen use within each sampled setting, at three levels of adaptability.

Setting	Adaptability	Conditional Effect of Screen Use	SE	t	*p*
Sichuan (n = 698)	Low (28)	0.836	0.075	11.22	<0.001
	Mean (37)	0.602	0.044	13.72	<0.001
	High (47)	0.341	0.073	4.66	<0.001
Yunnan (n = 701)	Low (28)	0.751	0.087	8.67	<0.001
	Mean (37)	0.344	0.056	6.12	<0.001
	High (47)	−0.107	0.060	−1.78	0.076

Note: Values are the 16th, 50th, and 84th percentiles of adaptability. The conditional interaction between screen use and adaptability was −0.026 in Sichuan and −0.045 in Yunnan. Because each setting is represented by a single province, these contrasts describe differences between the two sampled settings only.

## Data Availability

The data that support the findings of this study are available from the corresponding author upon reasonable request.

## Supplementary Materials

The following supporting information can be downloaded at: https://www.mdpi.com/article/10.3390/bs16101919/s1, Figure S1: Correlation heatmap of the study variables; Table S1: Data cleaning record: out-of-range values and item-level missingness; Table S2: Sampling record; Table S3: Descriptive statistics of the study variables (N = 1399); Table S4: Descriptive statistics of the individual items (51 items); Table S5: Reliability statistics (Cronbach’s alpha); Table S6: Item-total statistics for the five scales and for the 26-item total score; Table S7: Pearson correlations among the study variables (N = 1399); Table S8: Group statistics for academic burnout by sampled setting and gender; Table S9: Independent-samples tests on academic burnout; Table S10: Hierarchical regression predicting academic burnout: model summary; Table S11: Hierarchical regression predicting academic burnout: ANOVA (Model 3); Table S12: Hierarchical regression predicting academic burnout: coefficients (Model 3); Table S13: Conditional effect of screen use on academic burnout at values of psychological quality (total score); Table S14: Moderation of the screen-use-burnout association by each dimension of psychological quality; Table S15: Three-way interaction between screen use, adaptability and sampling setting: model summary; Table S16: Three-way interaction model: coefficients; Table S17. Conditional effect of screen use within each sampled setting, at three levels of adaptability; Table S18. Exploratory analysis: moderation by gender; Table S19. Exploratory factor analysis of the psychological quality items (principal component analysis, three components).

## Abbreviations

COR	Conservation of Resources Theory
ACM	Adaptive Calibration Model
PMUM-SF	Problematic Media Use Measure-Short Form
CI	Confidence interval
CMB	Common method bias
