# Supplementary material for: Does Psychological Quality Matter? A Moderated Model of Family Screen Exposure and Academic Burnout Among Early Adolescents
Source: Behav Sci (Basel). 2026 Oct 16;16(10):1919. doi: 10.3390/bs16101919 (PMC13701448; doi:10.3390/bs16101919)
Supplement: Supplementary file 1 [file behavsci-16-01919-s001.zip › behavsci-4580453-supplementary.pdf]

Supplementary Material

Statistical output underlying the Results section

Manuscript behavsci-4580453 · Does Psychological Quality Matter? A Moderated Model of Family Screen Exposure and Academic Burnout Among Early Adolescents

Note. All statistics in this document were computed from the analysis file (N = 1,399). Item names are the variable names in the analysis data file (PFSU = problematic family screen use; AB = academic burnout, items 4, 6, 8, 9, 13, 14 and 15 reverse-scored; CC = cognitive characteristics; PQ = personal qualities; AD = adaptability).

1. Data cleaning and the sampling record

Table S1. Data cleaning record: out-of-range values and item-level missingness.

Scale	Item	Out-of-range values replaced	Values imputed	% of cases
Problematic family screen use	PFSU1	0	19	1.4
	PFSU2	0	63	4.5
	PFSU3	0	7	0.5
	PFSU4	0	457	32.7
	PFSU5	0	504	36.0
	PFSU6	0	12	0.9
	PFSU7	0	12	0.9
	PFSU8	0	21	1.5
	PFSU9	0	456	32.6
Academic burnout	AB1	0	9	0.6
	AB2	0	7	0.5
	AB3	0	15	1.1
	AB4	0	14	1.0
	AB5	0	12	0.9
	AB6	0	21	1.5
	AB7	0	27	1.9
	AB8	0	21	1.5
	AB9	0	19	1.4
	AB10	0	9	0.6
	AB11	0	2	0.1
	AB12	0	14	1.0
	AB13	0	27	1.9
	AB14	0	18	1.3
	AB15	0	26	1.9
	AB16	0	24	1.7
Cognitive characteristics	CC1	1	13	0.9
	CC2	0	21	1.5
	CC3	0	8	0.6
	CC4	0	10	0.7
	CC5	0	5	0.4
	CC6	0	12	0.9
	CC7	0	6	0.4
Personal qualities	PQ1	0	5	0.4
	PQ2	0	7	0.5
	PQ3	1	4	0.3
	PQ4	0	11	0.8
	PQ5	0	8	0.6
	PQ6	0	11	0.8
	PQ7	0	14	1.0
	PQ8	0	19	1.4
Adaptability	AD1	2	3	0.2
	AD2	0	19	1.4
	AD3	0	16	1.1

Scale	Item	Out-of-range values replaced	Values imputed	% of cases
	AD4	1	22	1.6
	AD5	1	20	1.4
	AD6	1	58	4.1
	AD7	1	3	0.2
	AD8	0	4	0.3
	AD9	0	4	0.3
	AD10	0	15	1.1
	AD11	0	11	0.8
All items	51	8	2,145	3.0 (of 51 x 1,399 scores)

Note. Out-of-range values are responses outside the 1-5 response format; each was replaced by the mean of the valid responses to the same item. Twenty such values were found in the exported file, of which the 8 that fall on the analysis variables (across 7 items and 8 cases) are listed above. The fourth column gives the item-level values that were missing in the returned questionnaires and were imputed for the analysis file; in total 2,145 item scores (3.0% of 51 x 1,399) are of this kind. The three screen items PFSU4, PFSU5 and PFSU9 account for 1,417 of them (32.7%, 36.0% and 32.6% of cases respectively), whereas every other item is below 5% and the largest of the rest accounts for 4.5% of cases. All statistics in the manuscript and in this document were computed from the imputed analysis file, which contains no item-level missing values and no out-of-range values, and in which every case is complete on all 51 items and on the covariates.

Table S2. Sampling record.

Item	Value
Questionnaires distributed	1,500
Questionnaires set aside as incomplete or invalid	101
Complete and valid responses retained	1,399
Analysis sample (complete on all scales and covariates)	1,399
Setting 1 (Sichuan)	698
Setting 2 (Yunnan)	701
Fifth grade	642
Sixth grade	757
Boys	650
Girls	749
Classes surveyed in each school	two fifth-grade and three sixth-grade
Students per class	38 to 47
Schools sampled	not identified in the analysis file

Note. The analysis file carries no school or class identifier, so the number of schools cannot be recovered from it and the models do not adjust for clustering; this is noted as a limitation in Section 4.6 of the manuscript.

2. Descriptive statistics, reliability and correlations

Table S3. Descriptive statistics of the study variables (N = 1399).

Variable	N	Min	Max	M	SD	Variance	Skewness	SE	Kurtosis	SE
Problematic family screen use	1399	9.00	45.00	22.31	7.95	63.18	0.090	0.065	-0.912	0.131
Academic burnout	1399	23.00	80.00	49.86	11.63	135.29	0.478	0.065	-0.313	0.131
Cognitive characteristics	1399	11.00	35.00	27.15	5.06	25.61	-0.451	0.065	-0.357	0.131
Personal qualities	1399	13.00	40.00	31.38	5.48	29.98	-0.545	0.065	-0.051	0.131
Adaptability	1399	16.00	55.00	37.48	8.71	75.87	0.136	0.065	-0.910	0.131
Psychological quality (total)	1399	47.00	130.00	96.01	16.03	256.93	0.077	0.065	-0.523	0.131

Note. All variables are scale sums; higher scores indicate more of the construct named. The standard errors of skewness and kurtosis are sqrt(6/N) = 0.065 and sqrt(24/N) = 0.131 respectively.

Table S4. Descriptive statistics of the individual items (51 items).

**Item**	**N**	**Min**	**Max**	**M**	**SD**
PFSU1	1399	1	5	1.718	1.154
PFSU2	1399	1	5	1.871	1.212
PFSU3	1399	1	5	2.931	1.528
PFSU4	1399	1	5	2.651	1.285
PFSU5	1399	1	5	2.913	1.265
PFSU6	1399	1	5	2.658	1.552
PFSU7	1399	1	5	2.664	1.574
PFSU8	1399	1	5	1.895	1.234
PFSU9	1399	1	5	3.011	1.328
AB1	1399	1	5	3.606	1.163
AB2	1399	1	5	3.004	1.513
AB3	1399	1	5	2.579	1.457
AB4	1399	1	5	2.607	1.414
AB5	1399	1	5	2.355	1.480
AB6	1399	1	5	2.315	1.419
AB7	1399	1	5	3.128	1.364
AB8	1399	1	5	2.630	1.570
AB9	1399	1	5	3.117	1.495
AB10	1399	1	5	2.534	1.579
AB11	1399	1	5	3.099	1.468
AB12	1399	1	5	2.831	1.485
AB13	1399	1	5	2.010	1.242
AB14	1399	1	5	3.266	1.325
AB15	1399	1	5	2.528	1.460
AB16	1399	1	5	3.192	1.514
CC1	1399	1	5	3.545	1.321
CC2	1399	1	5	3.798	1.174
CC3	1399	1	5	3.934	1.116
CC4	1399	1	5	3.918	1.076
CC5	1399	1	5	3.951	1.064
CC6	1399	1	5	3.964	1.048
CC7	1399	1	5	4.038	1.081
PQ1	1399	1	5	3.870	1.098
PQ2	1399	1	5	4.160	0.952
PQ3	1399	1	5	3.932	1.081
PQ4	1399	1	5	3.951	1.084
PQ5	1399	1	5	3.969	1.036
PQ6	1399	1	5	4.013	1.107
PQ7	1399	1	5	3.909	1.060
PQ8	1399	1	5	3.580	1.200
AD1	1399	1	5	3.906	1.105
AD2	1399	1	5	3.398	1.583
AD3	1399	1	5	2.454	1.517
AD4	1399	1	5	3.101	1.567
AD5	1399	1	5	2.843	1.497
AD6	1399	1	5	3.275	1.421
AD7	1399	1	5	3.894	1.114
AD8	1399	1	5	3.856	1.101
AD9	1399	1	5	3.765	1.111
AD10	1399	1	5	3.412	1.339
AD11	1399	1	5	3.574	1.393

Note. Item names are the variable names in the analysis data file. All responses lie between 1 and 5. The item means of the nine screen-use items sum to 22.311, exactly the scale mean in Table 1; the item means of the sixteen burnout items sum to 49.856 after the seven positively worded items are reverse-scored, exactly the scale mean in Table 1.

Table S5. Reliability statistics (Cronbach's alpha).

Scale	Number of items	Cronbach's alpha
Problematic family screen use	9	0.830
Academic burnout	16	0.806
Cognitive characteristics	7	0.760
Personal qualities	8	0.788
Adaptability	11	0.808
Psychological quality (total)	26	0.885

Note. Alpha was computed on the same 1,399 cases used throughout this document.

Table S6. Item-total statistics for the five scales and for the 26-item total score.

Scale	Item	Scale mean if item deleted	Scale variance if item deleted	Corrected item-total correlation	Cronbach's alpha if item deleted
Problematic family screen use	PFSU1	20.593	54.917	0.405	0.826
Problematic family screen use	PFSU2	20.440	54.169	0.423	0.825
Problematic family screen use	PFSU3	19.380	47.416	0.638	0.800
Problematic family screen use	PFSU4	19.660	50.133	0.626	0.803
Problematic family screen use	PFSU5	19.398	56.550	0.265	0.841
Problematic family screen use	PFSU6	19.653	47.380	0.627	0.802
Problematic family screen use	PFSU7	19.647	46.626	0.655	0.798
Problematic family screen use	PFSU8	20.416	52.810	0.494	0.818
Problematic family screen use	PFSU9	19.299	48.825	0.679	0.797
Academic burnout	AB1	46.249	129.239	0.178	0.808
Academic burnout	AB2	46.851	118.480	0.441	0.793
Academic burnout	AB3	47.277	119.272	0.437	0.793
Academic burnout	AB4	46.462	125.752	0.238	0.806
Academic burnout	AB5	47.501	112.352	0.661	0.777
Academic burnout	AB6	46.170	116.366	0.552	0.785
Academic burnout	AB7	46.728	122.751	0.353	0.799
Academic burnout	AB8	46.486	128.396	0.125	0.816
Academic burnout	AB9	46.972	115.001	0.563	0.784
Academic burnout	AB10	47.322	111.991	0.623	0.779
Academic burnout	AB11	46.757	116.022	0.541	0.786
Academic burnout	AB12	47.024	117.809	0.474	0.790
Academic burnout	AB13	45.866	129.922	0.135	0.811
Academic burnout	AB14	47.122	120.557	0.446	0.793
Academic burnout	AB15	46.384	122.647	0.325	0.801
Academic burnout	AB16	46.663	120.624	0.372	0.798
Cognitive characteristics	CC1	23.603	18.368	0.485	0.731
Cognitive characteristics	CC2	23.350	18.921	0.520	0.721
Cognitive characteristics	CC3	23.213	19.455	0.499	0.726
Cognitive characteristics	CC4	23.229	19.545	0.516	0.723
Cognitive characteristics	CC5	23.197	20.835	0.375	0.751
Cognitive characteristics	CC6	23.183	19.678	0.520	0.722
Cognitive characteristics	CC7	23.109	20.179	0.439	0.739
Personal qualities	PQ1	27.515	24.087	0.435	0.774
Personal qualities	PQ2	27.224	24.931	0.436	0.773
Personal qualities	PQ3	27.452	23.796	0.476	0.767
Personal qualities	PQ4	27.434	23.458	0.509	0.762
Personal qualities	PQ5	27.415	23.609	0.526	0.759
Personal qualities	PQ6	27.372	22.815	0.562	0.753
Personal qualities	PQ7	27.475	23.232	0.550	0.755

Scale	Item	Scale mean if item deleted	Scale variance if item deleted	Corrected item-total correlation	Cronbach's alpha if item deleted
Personal qualities	PQ8	27.804	23.256	0.456	0.772
Adaptability	AD1	33.573	67.786	0.378	0.801
Adaptability	AD2	34.080	57.680	0.653	0.771
Adaptability	AD3	35.024	65.446	0.331	0.808
Adaptability	AD4	34.377	58.237	0.635	0.773
Adaptability	AD5	34.635	62.398	0.475	0.792
Adaptability	AD6	34.203	61.883	0.535	0.785
Adaptability	AD7	33.584	67.845	0.370	0.801
Adaptability	AD8	33.622	69.095	0.304	0.806
Adaptability	AD9	33.713	70.362	0.229	0.812
Adaptability	AD10	34.066	61.674	0.590	0.780
Adaptability	AD11	33.904	60.620	0.614	0.777
Psychological quality (total)	CC1	92.465	233.688	0.532	0.878
Psychological quality (total)	CC2	92.212	236.127	0.539	0.878
Psychological quality (total)	CC3	92.076	238.969	0.484	0.880
Psychological quality (total)	CC4	92.092	239.466	0.490	0.880
Psychological quality (total)	CC5	92.059	243.641	0.366	0.882
Psychological quality (total)	CC6	92.046	237.323	0.574	0.878
Psychological quality (total)	CC7	91.972	240.544	0.454	0.880
Psychological quality (total)	PQ1	92.140	238.711	0.502	0.879
Psychological quality (total)	PQ2	91.850	245.508	0.353	0.883
Psychological quality (total)	PQ3	92.078	242.058	0.408	0.881
Psychological quality (total)	PQ4	92.059	242.069	0.406	0.881
Psychological quality (total)	PQ5	92.041	240.285	0.485	0.880
Psychological quality (total)	PQ6	91.997	239.216	0.482	0.880
Psychological quality (total)	PQ7	92.101	237.929	0.547	0.878
Psychological quality (total)	PQ8	92.430	238.026	0.471	0.880
Psychological quality (total)	AD1	92.104	237.182	0.545	0.878
Psychological quality (total)	AD2	92.612	231.595	0.474	0.880
Psychological quality (total)	AD3	93.556	247.625	0.147	0.890
Psychological quality (total)	AD4	92.909	232.134	0.468	0.880
Psychological quality (total)	AD5	93.167	242.749	0.256	0.886
Psychological quality (total)	AD6	92.735	237.677	0.393	0.882
Psychological quality (total)	AD7	92.116	236.218	0.569	0.878
Psychological quality (total)	AD8	92.154	238.272	0.513	0.879
Psychological quality (total)	AD9	92.245	239.919	0.458	0.880
Psychological quality (total)	AD10	92.598	233.656	0.525	0.878
Psychological quality (total)	AD11	92.436	234.206	0.488	0.879

Note. The corrected item-total correlations of Table 3 of the manuscript are the minima and maxima of the corresponding columns above: screen use 0.27-0.68, academic burnout 0.13-0.66, cognitive characteristics

0.38-0.52, personal qualities 0.44-0.56, adaptability 0.23-0.65, 26-item total 0.15-0.57. The seven reverse-scored burnout items are items 4, 6, 8, 9, 13, 14 and 15.

Table S7. Pearson correlations among the study variables (N = 1399).

Variable	1	2	3	4	5	6
1. Problematic family screen use	1	0.453***	-0.358***	-0.271***	-0.565***	-0.513***
2. Academic burnout		1	-0.005	0.129***	-0.513***	-0.236***
3. Cognitive characteristics			1	0.758***	0.504***	0.849***
4. Personal qualities				1	0.409***	0.803***
5. Adaptability					1	0.842***
6. Psychological quality (total)						1

Note. *** p < 0.001 (two-tailed). The correlation between cognitive characteristics and academic burnout (-0.005) is not significant (p = 0.841). Correlations among the three dimensions range from 0.41 to 0.76 and their correlations with the total score range from 0.80 to 0.85.

	1. PFSU	2. Academic burnout	3. Cognitive characteristics	4. Personal qualities	5. Adaptability	6. Psychological quality (total)
1. PFSU	1.00	0.45	-0.36	-0.27	-0.56	-0.51
2. Academic burnout		1.00	-0.01	0.13	-0.51	-0.24
3. Cognitive characteristics			1.00	0.76	0.50	0.85
4. Personal qualities				1.00	0.41	0.80
5. Adaptability					1.00	0.84
6. Psychological quality (total)						1.00

Figure S1. Correlation heatmap of the six study variables (N = 1,399). Cell values are Pearson correlations; the same values are tabulated in Table S7.

3. Group comparisons

Table S8. Group statistics for academic burnout by sampled setting and gender.

Grouping variable	Level	N	Mean	SD	Std. error mean
Sampled setting	Sichuan	698	46.56	12.18	0.461
Sampled setting	Yunnan	701	53.14	10.04	0.379
Gender	Boys	650	49.91	11.31	0.444
Gender	Girls	749	49.81	11.91	0.435

Note. The grouping variables are coded 1 = Sichuan, 2 = Yunnan and 0 = boys, 1 = girls in the analysis file.

Table S9. Independent-samples tests on academic burnout.

Comparison	Levene F	Levene p	t	df	p (2-tailed)	Mean difference	SE difference	95% CI	Cohen's d
Sichuan vs Yunnan (equal variances assumed)	17.892	< 0.001	-11.036	1397	< 0.001	-6.585	0.597	[-7.756, -5.415]	-0.590
Sichuan vs Yunnan (equal variances not assumed)			-11.032	1345.754	< 0.001	-6.585	0.597	[-7.756, -5.414]	
Boys vs Girls (equal variances assumed)	2.472	0.116	0.161	1397	0.872	0.100	0.624	[-1.123, 1.324]	0.009
Boys vs Girls (equal variances not assumed)			0.161	1385.689	0.872	0.100	0.621	[-1.119, 1.319]	

Note. Levene's test used the mean-centred form (SPSS default).

4. Moderation by psychological quality

Table S10. Hierarchical regression predicting academic burnout: model summary.

Model	R	R^2	Adjusted R^2	Std. error of the estimate	ΔR^2	ΔF	df1	df2	Sig. ΔF
1 (covariates)	0.327	0.107	0.105	11.0048	—	—	—	—	—
2 (+ main effects)	0.589	0.347	0.344	9.4178	0.2400	511.758	2	1392	< 0.001
3 (+ interaction)	0.593	0.351	0.348	9.3893	0.0044	9.474	1	1392	0.002

Note. Model 1 enters the covariates (setting, grade, gender); Model 2 adds the centred main effects of screen use and psychological quality; Model 3 adds their product. N = 1,399.

Table S11. Hierarchical regression predicting academic burnout: ANOVA (Model 3).

Source	Sum of squares	df	Mean square	F	Sig.
Regression	66,420.676	6	11,070.113	125.571	< 0.001
Residual	122,716.158	1392	88.158		
Total	189,136.833	1398			

Note. Dependent variable: academic burnout.

Table S12. Hierarchical regression predicting academic burnout: coefficients (Model 3).

Predictor	B	SE	Beta	t	Sig.	95% CI	VIF
Setting (1 = Sichuan, 2 = Yunnan)	9.5601	0.5458	0.411	17.515	< 0.001	[8.4903, 10.6300]	1.182
Grade	-4.2128	0.5340	-0.181	-7.889	< 0.001	[-5.2594, -3.1661]	1.124
Gender (0 = boys, 1 = girls)	-0.1795	0.5060	-0.008	-0.355	0.723	[-1.1713, 0.8122]	1.011
Screen use (centred)	0.6619	0.0371	0.452	17.836	< 0.001	[0.5892, 0.7347]	1.380
Psychological quality (centred)	-0.0267	0.0190	-0.037	-1.403	0.161	[-0.0639, 0.0106]	1.472
Screen use × Psychological quality	0.0069	0.0022	0.073	3.078	0.002	[0.0025, 0.0113]	1.207

Note. Screen use and psychological quality were mean-centred before the product term was computed. The interaction is positive, so the association is stronger at higher levels of the composite score, although the increment is small ($\Delta R^2 = 0.004$).

Table S13. Conditional effect of screen use on academic burnout at values of psychological quality (total score).

Value of the moderator	Conditional effect	SE	t	Sig.	95% CI
80 (16th/50th/84th percentile)	0.552	0.0548	10.070	< 0.001	[0.444, 0.659]
95 (16th/50th/84th percentile)	0.655	0.0375	17.481	< 0.001	[0.582, 0.728]
114 (16th/50th/84th percentile)	0.786	0.0512	15.352	< 0.001	[0.686, 0.886]

Note. Values are the 16th, 50th and 84th percentiles of the total score (the mean is 96.01 and the SD is 16.03). The conditional effect is the slope of screen use at that value.

Table S14. Moderation of the screen-use-burnout association by each dimension of psychological quality.

Moderator	Interaction B	SE	t	Sig.	95% CI	ΔR^2	ΔF (1, 1392)
Psychological quality (total)	0.0069	0.0022	3.078	0.002	[0.0026, 0.0112]	0.0044	9.47
Cognitive characteristics	0.0655	0.0062	10.563	< 0.001	[0.0533, 0.0777]	0.0472	111.58
Personal qualities	0.0587	0.0053	10.994	< 0.001	[0.0483, 0.0691]	0.0486	120.87
Adaptability	-0.0445	0.0039	-11.498	< 0.001	[-0.0521, -0.0369]	0.0467	132.20

Note. Each dimension was entered in a separate model together with setting, grade and gender and the corresponding centred main effects. ΔR^2 is the increment attributable to the product term.

5. Moderation by the sampled setting

Table S15. Three-way interaction between screen use, adaptability and sampling setting: model summary.

Statistic	Value
R	0.722
R^2	0.521
Adjusted R^2	0.518
Std. error of the estimate	8.0764
F (df1, df2)	167.848 (9, 1389)
Sig.	< 0.001
ΔR^2 for the three-way term	0.0019
ΔF (1, 1389) for the three-way term	5.425
Sig. ΔF	0.020

Note. Dependent variable: academic burnout. The ΔR^2 row compares this model with the same model without the product of screen use, adaptability and setting.

Table S16. Three-way interaction model: coefficients.

Predictor	B	SE	t	Sig.	95% CI	VIF
Screen use (centred)	0.8554	0.1037	8.250	< 0.001	[0.6522, 1.0586]	14.557
Adaptability (centred)	-0.7832	0.1046	-7.487	< 0.001	[-0.9882, -0.5781]	17.795
Screen use × Adaptability	-0.0070	0.0136	-0.511	0.610	[-0.0336, 0.0197]	16.016
Setting (1 = Sichuan, 2 = Yunnan)	7.6729	0.5533	13.866	< 0.001	[6.5883, 8.7574]	1.642
Screen use × Setting	-0.2663	0.0707	-3.767	< 0.001	[-0.4049, -0.1278]	18.029
Adaptability × Setting	0.0849	0.0645	1.317	0.188	[-0.0415, 0.2112]	20.258
Screen use × Adaptability × Setting	-0.0191	0.0082	-2.329	0.020	[-0.0352, -0.0030]	19.177
Grade	-2.7351	0.4749	-5.759	< 0.001	[-3.6659, -1.8042]	1.201
Gender (0 = boys, 1 = girls)	-0.7573	0.4351	-1.741	0.082	[-1.6101, 0.0954]	1.010

Note. The sampled setting was coded 1 = Sichuan and 2 = Yunnan. Because the setting is binary, the lower-order terms that involve it refer to a setting value of zero, which lies outside the observed range; they are used only to construct the conditional effects reported below. The variance inflation factors of the product terms are high, which is expected in a model that contains a three-way interaction and does not by itself indicate an estimation problem.

Table S17. Conditional effect of screen use within each sampled setting, at three levels of adaptability.

Sampled setting	Adaptability	Conditional effect	SE	t	Sig.
Sichuan	28 (16th/50th/84th pct.)	0.8361	0.0745	11.216	< 0.001
	37 (16th/50th/84th pct.)	0.6015	0.0438	13.724	< 0.001
	47 (16th/50th/84th pct.)	0.3409	0.0732	4.656	< 0.001
Yunnan	28 (16th/50th/84th pct.)	0.7509	0.0866	8.670	< 0.001
	37 (16th/50th/84th pct.)	0.3444	0.0562	6.123	< 0.001
	47 (16th/50th/84th pct.)	-0.1073	0.0603	-1.778	0.076

Note. Conditional effects are the slope of screen use on academic burnout at the 16th, 50th and 84th percentiles of adaptability within each setting, evaluated from the coefficients in Table S16. The conditional interaction between screen use and adaptability was -0.026 in Sichuan ($F = 17.25$, $p < 0.001$) and -0.045 in Yunnan ($F = 73.16$, $p < 0.001$).

6. Exploratory analyses

Table S18. Exploratory analysis: moderation by gender.

Predictor	B	SE	t	Sig.	95% CI
Screen use (centred)	0.7022	0.0473	14.856	< 0.001	[0.6095, 0.7948]
Gender (0 = boys, 1 = girls)	-0.1801	0.5064	-0.356	0.722	[-1.1727, 0.8125]
Screen use × Gender	0.0284	0.0638	0.446	0.656	[-0.0966, 0.1535]
Setting (1 = Sichuan, 2 = Yunnan)	9.1339	0.5376	16.991	< 0.001	[8.0803, 10.1876]
Grade	-4.1832	0.5365	-7.797	< 0.001	[-5.2347, -3.1316]

Note. Reported for completeness; no directional hypothesis was advanced for gender. ΔR^2 for the product term = 0.001, $\Delta F(1, 1393) = 0.98$, $p = 0.322$. The simple slope of screen use is 0.662 for boys and 0.732 for girls.

Table S19. Exploratory factor analysis of the psychological quality items (principal component analysis, three components).

Item	Dimension	Factor 1	Factor 2	Factor 3	Communality
AD1	adaptability	0.627	0.031	0.008	0.394
AD2	adaptability	0.408	0.713	0.208	0.719
AD3	adaptability	0.070	0.528	0.638	0.691
AD4	adaptability	0.413	0.635	0.055	0.576
AD5	adaptability	0.164	0.660	0.395	0.618
AD6	adaptability	0.333	0.646	0.237	0.584
AD7	adaptability	0.656	0.070	0.142	0.455
AD8	adaptability	0.607	0.140	0.318	0.489
AD9	adaptability	0.570	0.201	0.005	0.365
AD10	adaptability	0.488	0.508	0.071	0.501
AD11	adaptability	0.442	0.595	0.102	0.560
CC1	cognitive	0.574	0.177	0.181	0.394
CC2	cognitive	0.617	0.023	0.064	0.385
CC3	cognitive	0.591	0.147	0.185	0.405
CC4	cognitive	0.579	0.048	0.235	0.393
CC5	cognitive	0.492	0.317	0.180	0.375
CC6	cognitive	0.674	0.118	0.037	0.470
CC7	cognitive	0.538	0.053	0.253	0.357
PQ1	personal	0.565	0.042	0.082	0.328
PQ2	personal	0.471	0.281	0.143	0.321
PQ3	personal	0.524	0.276	0.246	0.411
PQ4	personal	0.538	0.337	0.161	0.428
PQ5	personal	0.604	0.241	0.162	0.450
PQ6	personal	0.600	0.266	0.208	0.474
PQ7	personal	0.663	0.178	0.164	0.498
PQ8	personal	0.547	0.022	0.041	0.301

Note. Three components had eigenvalues greater than 1 (7.377, 3.326 and 1.239) and together accounted for 45.93% of the variance (28.37%, 12.79% and 4.76%). All seven cognitive and all eight personal items load on the first component, whereas the adaptability items split across the three components. Absolute loadings are shown. This supports the caution in Section 4.3 of the manuscript against dimension-level interpretation of this instrument.
